# Occult Infection with Hepatitis C Virus: Looking for Clear-Cut Boundaries and Methodological Consensus

**DOI:** 10.3390/jcm10245874

**Published:** 2021-12-14

**Authors:** Anna Wróblewska, Krzysztof Piotr Bielawski, Katarzyna Sikorska

**Affiliations:** 1 Laboratory of Photobiology and Molecular Diagnostics, Intercollegiate Faculty of Biotechnology, University of Gdansk and Medical University of Gdansk, Abrahama 58, 80-307 Gdansk, Poland; anna.wroblewska@ug.edu.pl (A.W.); krzysztof.bielawski@ug.edu.pl (K.P.B.); 2 Department of Tropical Medicine and Epidemiology, Faculty of Health Sciences, Institute of Maritime and Tropical Medicine, Medical University of Gdansk, Powstania Styczniowego 9b, 81-519 Gdynia, Poland

**Keywords:** occult hepatitis C infection, extrahepatic complications, lymphotropism, viral persistence, HCV-RNA detection

## Abstract

The sustained virologic response and elimination of HCV is widely viewed as a true cure of chronic hepatitis C as it associates with amelioration of histological liver damage and improved clinical outcomes. Therefore, the existence and clinical burden of occult HCV infection (OCI) has been a controversial issue for many years. In this review, we summarize recently published data that adds new information on the molecular and clinical background of OCI and its epidemiological significance. We also identify and discuss the most important methodological pitfalls, which can be a source of inconsistency between studies. Data that have accumulated so far, strongly support the existence of extrahepatic HCV replication in individuals negative for serum HCV-RNA by conventional clinical tests. OCI emerges as a condition where the immune system is unable to fully resolve infection but it is continuously stimulated by low levels of HCV antigens, leading to progression of liver pathology and extrahepatic HCV-related complications. Moreover, the development of monitoring strategies or management guidelines for OCI is still hampered by the lack of clear definition and the confusion regarding its clinical significance. Careful study design and the introduction of uniform protocols for the detection of low-level HCV-RNA are crucial for obtaining reliable data on OCI.

## 1. Introduction

It is estimated that 58 million people globally have chronic hepatitis C virus infection (CHC) and 1.5 million new infections occur every year. Treatment schemes with direct-acting antivirals (DAA) display over 95% effectiveness in HCV eradication and the number of treated patients is increasing rapidly, following improvements in worldwide accessibility of these drugs [1]. The therapeutic end-point for DAA therapy is currently recognized as the sustained virological response (SVR) in serum assessed at 12 weeks after the end of treatment using RNA nucleic acid testing (NAT) technologies with a lower limit of detection (LoD) of 15 IU/mL. Despite successful HCV clearance, some patients fail to experience improvement in clinical and immunological parameters. The risk of HCC recurrence or clinical relapse of extrahepatic HCV complications still exists after therapeutically induced SVR [2].

Beginning from 2004, independent research groups started to find small amounts of HCV-RNA in liver tissue, serum, and in peripheral blood mononuclear cells (PBMCs) of patients, who were never diagnosed with HCV infection or who achieved therapeutically induced SVR [3,4,5]. Since then, a widely accepted definition of occult HCV infection (OCI) has been developed. It has been defined by the presence of HCV-RNA in hepatocytes, and/or PBMCs in individuals, who are HCV-RNA negative in serum by conventional diagnostic tests (with LoD 15 IU/mL). Depending on the presence or absence of anti-HCV in serum, two types of OCI are distinguished—seronegative and seropositive [6]. Occult viral persistence by definition escapes clinical diagnostic schemes and can last for many years after spontaneous or treatment-induced SVR. However, its true clinical and epidemiological impact remains to be elucidated.

The aim of this review was to summarize the most recent data on the molecular mechanisms of OCI, as well as its clinical and epidemiological significance. Our goal was also to give an overview of the methodological background of current diagnosis of OCI in order to show the potential sources of inconsistency between reports. PubMed was searched for the latest English-language articles on these subjects. Additionally, several of the most recent articles with research on OCI prevalence were selected to underline methodological differences and inconsistencies in reporting. This data served to determine suggested methodological key points to be considered in research on OCI.

## 2. Consequences of HCV Lymphotropism

Hepatitis C virus, although mainly hepatotropic, is also able to infect and proliferate in other tissues and organs such as gastrointestinal mucosa [7], lymph nodes [8], bone marrow [9], and all the main subsets of circulating lymphoid cells, including T cells, B cells, and monocytes [3,10,11,12,13]. HCV infection is linked with the existence of phylogenetically distinct viral quasispecies in different tissue and cellular compartments. This compartmentalization is of key importance for HCV evolution within a host and the natural history of the infection. Different replication dynamics in various compartments lead to a very high evolutionary heterogeneity that, together with a strong HCV population structure, contributes to viral persistence [14].

Analyses of lymphotropic HCV variants showed differences in internal ribosome entry site (IRES), a highly conserved RNA structure essential for translation of the viral polyprotein, located at the 5′ untranslated region (5′-UTR), and in hypervariable region-1 of viral E2 protein in comparison to the HCV genomes from plasma and liver, reflecting viral adaptation to propagation in lymphoid cells [15,16]. Additionally, polymorphisms in IRES sequence of lymphoid variants corresponded with different IRES translational activity. For example, HCV IRES variants from plasma displayed higher translational efficiency than those found in B cells. B-cell-specific IRES sequences exhibited low translational activity in hepatoma Huh7 cell line and primary human hepatocytes, but not in B cell lines [15]. Importantly, HCV replicating in lymphoid cells, but not in the liver, could have a major contribution to the total level of viremia. It was shown that 68% of HCV variants circulating in serum were also present in lymphoid tissues, and only 40% of serum quasispecies originated from the liver [8]. Lymphatic variants of HCV localized in PBMC were also detectable in serum but not in liver tissue [17]. Some studies also suggest that infected monocytes/macrophages can transmit the virus into the central nervous system where HCV replication is maintained by microglial cells [18]. Analysis of HCV-RNA sequences showed that from 24 up to 55% of brain-derived variants were absent from the serum, but at the same time they were identical to HCV-RNA sequences found in the lymph nodes [16,18].

The direct interaction of HCV with lymphoid cells induces profound changes in their functions. Infection with HCV was shown to induce immunosenescence and T cell exhaustion, promote immune cell apoptosis, and stimulate carcinogenesis. In particular, it was shown that cellular topoisomerase I from T cells of CHC patients is significantly inhibited, which promotes distortion of DNA topology leading to T cell senescence and apoptosis [19]. Inhibition of T cell proliferation and induction of Fas-mediated apoptosis was also shown in established T cell lines [20]. HCV infection in CD4+ T cells was found not only to disrupt proliferation but also to inhibit IFN-γ production by these cells [21] and to distort their differentiation towards Th1 lineage [22]. Interestingly proliferation of CD4+ cells was inhibited by exposure to the exogenous HCV particles, even without molecularly evident viral replication within the cells [22]. Viral E2 protein and E2 protein-encoding RNA were shown to inhibit T cell receptor signaling, which is crucial for differentiation, activation, proliferation, and effector functions of CD4+ and CD8+ cells [23]. HCV core protein was able to transcriptionally modulate IL-2 promoter [24] and upregulate programmed cell death-1 protein expression in T cells [25]. Additionally, it was found that chronic HCV infection favors the development of Th17 type response, which plays a role in autoimmune diseases [26]. Infection with HCV is a known risk factor of mixed cryoglobulinemia and lymphoproliferative disorders, particularly B-cell non-Hodgkin lymphoma. The underlying mechanism includes prolonged antigen stimulation and pathological proliferation of B cells. Nonstructural HCV protein NS3/4A upregulates B-cell receptor signaling (BCR), a pathway involved in B-cell maturation and activation, critical for the development of several B-cell lymphomas [27]. HCV E2 protein can also directly stimulate B cell activation, omitting BCR signaling, by interaction with CD81 receptor on B-cells [28] Additionally, HCV infection is known to enhance mutation frequency in B cells thereby inducing oncogenesis [29]. It was shown that the expression of certain genes involved in lymphoma development increases in B cells of patients after IFN therapy-induced SVR, and this is more pronounced when the residual HCV can be detected in PBMC [30]. Chronic B cell stimulation in CHC results in expansion of atypical autoreactive CD27+IgM+CD21low/− memory B cells that associate with HCV-induced lymphoproliferation and cryoglobulinemia [31].

## 3. Mechanisms of Occult HCV Persistence

The virus has evolved many mechanisms that enable immune escape and lead to immunological exhaustion of the host. Acute HCV infection in the majority (55–85%) of cases develops into a chronic condition when both HCV-RNA and anti-HCV antibodies can be detected in serum. There are also other scenarios including progression of HCV infection without detectable HCV-RNA in seronegative individuals. The outcome of the infection is the result of virological factors (e.g., route of infection, viral inoculum, and HCV genotype), immunological competence of the host, and impact of antiviral therapy [32]. The exact molecular events that determine viral persistence in patients with low or undetectable viremia in serum remain unknown. There are reports that suggest that HCV responds to increased immune pressure by switching to a latent state of infection enabling viral survival within the host.

Viral double-stranded RNA (dsRNA) is a known pathogen-associated molecular pattern and a potential HCV reservoir. During chronic infection it comprises around half of HCV-RNA present in patients’ livers and its de novo formation is stimulated by IFN treatment [33]. During OCI, HCV resides inside cells mainly in the form of dsRNA, as the proportions of HCV-RNA strand (+) and HCV-RNA (−) were found to be close to 1 [34]. This is in contrast with the liver of CHC patients where the level of HCV-RNA (−) strand is 1 to 2 logs lower than the level of (+) strand [35]. These data suggest that a very low level of replication and a deficient virion synthesis occurs in OCI. Such differences in replication dynamics of HCV have also been observed within the various organ and cellular compartments in chronically infected patients as the ratio of HCV-RNA (−) to (+) strand was much lower in macrophages than in corresponding liver tissue samples [35].

Other observations link the persistence of HCV with increased immunological pressure related to intrinsic properties of the host immunity and antiviral treatment. Genotype TT in IL28 rs12979860 polymorphism is associated with increased cellular IFN signaling, elevated expression levels of IFN-stimulated genes, and a non-response to treatment in CHC patients [36]. Interestingly this genotype correlated also with a higher percentage of dsRNA in liver tissue in CHC patients [33] and associated with the occurrence of both seropositive and seronegative OCI [34,37,38,39]. Analysis of 5′UTR variants emerging during therapy with peg-IFN and ribavirin showed that the majority (64%) of the new variants were localized in PBMC and all had decreased IRES stability and impaired translational efficiency. Development of new replication-deficient lymphotropic variants could indicate viral adaptation to treatment-induced immune pressure, leading to decreased expression of viral proteins and facilitating viral persistence [40].

## 4. Immune Landscape of OCI

Patients with OCI exhibit distinct immune profiles from individuals who reached spontaneous or treatment-induced SVR, patients with CHC, and healthy controls. In comparison with healthy individuals, OCI patients show elevated expression of IFN-α, pro-inflammatory cytokine interferon gamma-induced protein 10 (IP-10), and Myxovirus resistance protein 1 (MxA) [41]. Additionally, occult HCV infection is associated with higher expression levels of IL-6, IL-8, IL-12, TNF-α, and macrophage inflammatory protein 1b compared to SVR individuals [42]. It was suggested that immunity skewed to Th2 type response is associated with HCV persistence. Indeed, CHC patients exhibit elevated expression of IL-10, an immunosuppressive cytokine, in their PBMC in comparison to individuals with self-limited hepatitis. Additionally, both in CHC and OCI high expression of IL-10 was accompanied by low or undetectable levels of serum IL-12 and IFN-γ, cytokines involved in T-cell mediated immunity and viral clearance [41]. Patients with seropositive OCI and those with CHC had significantly higher serum levels of Th1 (IL-2, IFN-γ) and Th2 (IL-4, IL-10) type cytokines in comparison with healthy subjects. However, patients with occult infection had even lower levels of IL-2 and IFN-γ as well as elevated concentrations of IL-4 and IL-10 when compared to chronically infected individuals [43]. The differences in immune setting between OCI and CHC is further depicted by the results of ex vivo stimulation of PBMC with mitogens [10]. When lymphatic viral load is low or undetectable as found in OCI HCV replication is augmented. On the other hand, in CHC, or sporadic cases of OCI with high PBMC viral load, ex vivo mitogen stimulation leads to suppression of replication. Additionally, viral replication after mitogen treatment inversely correlated with the intracellular level of IFN-α mRNA, but not IFN-γ or TNF- α expression, which suggests that IFN-α pathways play a key role in controlling viral replication during OCI [44].

**Figure 1:**
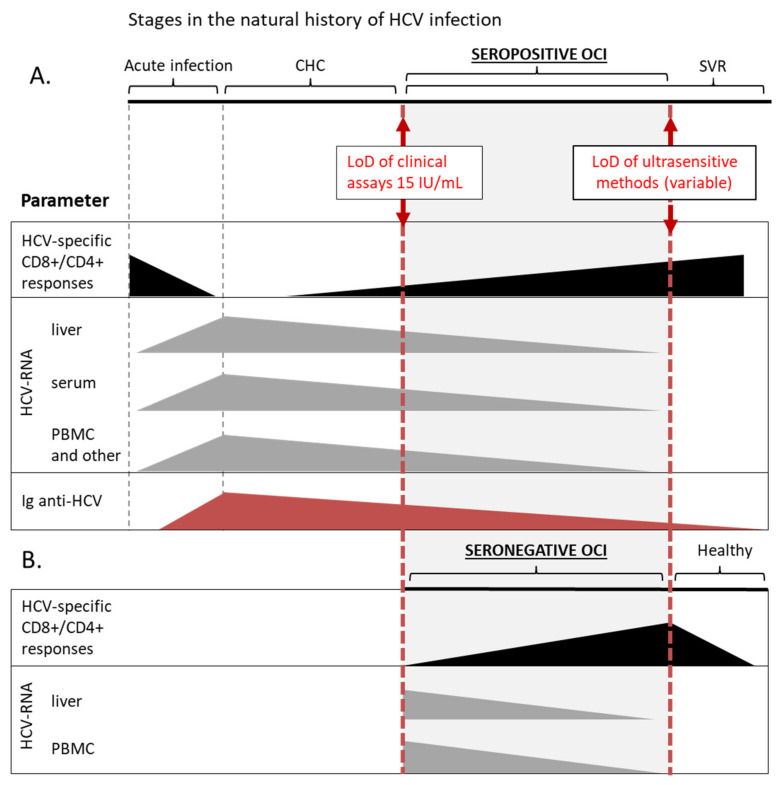
Schematic overview of OCI as one of the stages in the natural history of HCV infection. This chart summarizes data on the detection of basic parameters at different stages of HCV infection, including seropositive (**A**) and seronegative (**B**) OCI. Solid boxes represent a range of relative values of a particular parameter, that can be detected with current methods, and are not organized as a timeline. HCV-specific CD8+/CD4+ responses are marked with black color, the presence of HCV-RNA in different compartments with grey, and red triangles represent the level of anti-HCV immunoglobulins. In a patient with certain diagnosis different parameters can assume various values within the range, e.g., in a patient with seropositive OCI HCV-RNA can be detected in PBMC and not in the serum. LoD, lower limit of detection; CHC, chronic hepatitis C; SVR, sustained virological response; PBMC, peripheral blood mononuclear cells.

It was shown that HCV-specific CD4+ and CD8+ cellular immune responses exist in PBMC of seropositive patients with OCI and in 52% seronegative and aviremic individuals with HCV-RNA persisting in liver tissue [45,46] (Figure 1). These responses were more frequent and the numbers of reactive cells were higher in seronegative OCI than in CHC and cryptogenic liver disease [45]. The magnitude of T-cell reactivity inversely correlated with HCV-RNA level in patients’ liver tissue [45]. On the other hand, in seropositive OCI HCV-specific T-cell responses were lower than in SVR individuals but indistinguishable from those in CHC patients [46]. Additionally, the expression of low-density lipoprotein receptors, involved in HCV entry, was similar in lymphocytes and monocytes of OCI and CHC patients [47].

Antiviral therapy with DAAs induces profound changes in immune balance and was shown to evoke a general inflammatory state [48,49,50]. Reaching SVR generally leads to improvement in immune cell functions and homeostasis as well as amelioration of extrahepatic complications and this effect is inversely correlated with the severity of the disease before therapy [2]. Likewise, a complete viral clearance or occult HCV persistence after DAA seems to be determined by the patient’s immune responsiveness at baseline and by the individual ability to induce a general inflammatory response after viral suppression by DAAs [51,52]. In our study, a significant and transient elevation of neutrocyte-to-lymphocyte ratio, a marker of systemic inflammation, which occurred during and shortly after DAA therapy, predicted elimination of the replicating residual HCV in PBMC at the follow up after SVR [53]. Additionally, patients without HCV-RNA (−) strand showed a greater increase in neutrocyte counts during therapy [53]. Many pretreatment parameters are also linked with OCI occurrence after SVR. High viral load and ALT level, prolonged prothrombin time, low albumin, Child B score, raised bilirubin, and treatment experience predisposed to OCI after DAA therapy [54]. Basal liver necroinflammation and fibrosis scores were higher for patients who were later diagnosed with OCI after reaching treatment-induced SVR [55]. Moreover, as mentioned above, polymorphisms within immune-related genes such as chemokine ligand CXCL10 [56] or IL28 [34,37] determine viral persistence. In seronegative patients with hemophilia or kidney disease, OCI is linked with elevated biochemical parameters associated with systemic inflammation, such as cholesterol, triglyceride, low-density lipoprotein, and C-reactive protein level as well as with 25-hydroxyvitamin D deficiency [37,39]. These data show that OCI may actually reflect specific characteristics of the host immune system, including low-level inflammation and reduced responsiveness to stimulation, which allows the virus to persist even after apparent therapeutically-induced resolution of viremia. It remains to be studied what are the exact determinants of immunological dysfunction associated with occult viral persistence.

Taken together, it is now established that the virus replicating at low levels in PBMC is not neutral to the immune system but instead continues to stimulate and modulate immunological functions long after apparent viral clearance. The picture of OCI that emerges from these data suggest that it is a condition in-between chronic infection and a state of sustained response, where the immune system is able to restrict viral replication but the cellular immunity is still impaired and unable to completely clear the virus. The term ‘occult infection’ actually covers a range within the natural history of HCV infection, being, by definition, arbitrary restricted by the limits of methods for HCV-RNA detection (Figure 1). Nevertheless, OCI appears to be a separate disease entity distinct from both CHC and acute HCV infection, still lacking a complete description and uniform diagnostic criteria.

## 5. Clinical Consequences of OCI

The sustained virologic response and elimination of HCV from serum is viewed as a true cure of CHC, and it associates with the improvement of liver histology and significantly better clinical outcomes, even in patients with advanced liver fibrosis [57]. In comparison to CHC OCI is considered to be a milder form of infection with a smaller number of infected hepatocytes, associated with less pronounced hepatic injury, as well as more sporadic extrahepatic complications [58]. However, there are studies that show that there is still an association between OCI and hepatic necroinflammation or liver fibrosis both in treated CHC patients who reached SVR [55,58] and in those with seronegative OCI [4,59]. The persistence of residual hepatic HCV-RNA is frequent among patients with cryptogenic liver disease [4,60,61,62], which suggests that occult hepatitis C virus infection can be the underlying cause of liver damage. There is also evidence that increased risk of HCC still exists after therapeutically induced SVR [63] and that the residual amounts of virus present in the liver contributing to a prolonged liver injury can stimulate carcinogenesis [64,65,66].

Further confirmation of the association of OCI with liver pathology came from a study on an animal model of Hepacivirus infection. This report clearly showed that occult re-infection induces liver damage with hepatic lymphocyte infiltration and fibrosis, even in the absence of robust viral replication and in the presence of specific antiviral T-cell responses [67]. Wang et al. analyzed groups of 60 patients treated with DAAs, 50 with pegylated IFN, and a group of 30 individuals who spontaneously resolved HCV infection [55]. They tested PBMC samples and paired liver biopsies from treated patients for the presence of OCI using qPCR and RNAscope in situ hybridization assay, respectively. These authors unequivocally showed that OCI is positively associated with a higher degree of hepatic fibrosis and necroinflammation after treatment and that significant post-therapeutic regression of liver fibrosis occurs only in non-OCI patients, and not in individuals with OCI. Another interesting observation was a higher incidence of OCI among patients treated with IFN-free regimens in comparison to individuals treated with IFN, and to those who spontaneously resolved infection [55]. Although all patients included in this study were ethnically Chinese and the results need to be confirmed on larger groups of other human subpopulations, this work underlines the clinical significance of OCI and the need for post-treatment monitoring of CHC patients.

Existing clinical data show a link between OCI and extrahepatic HCV-related diseases such as glomerulonephritis, cryoglobulinemia, or lymphoproliferative disorders. Specifically, occult infection was found in 34 of 87 seronegative, aviremic patients with autoimmune-mediated glomerulonephritis as compared to 1 OCI case among 26 patients from the control group with hereditary glomerulonephritis [68]. The presence of OCI in these patients correlated with worse kidney function and faster progression to end-stage kidney disease in comparison to HCV-RNA negative individuals [68]. It was also shown that viral antigen NS3 and HCV particles are present in kidney tissue of seropositive and seronegative patients with glomerulonephritis, being likely the cause of renal injury [69,70,71]. Likewise, occult HCV infection may be a hidden cause of many cases of mixed cryoglobulinemia in patients with no evidence of past or present HCV infection [72,73]. Replicative HCV-RNA strand was found in PBMC of five out of nine patients with mixed cryoglobulinemia who reached SVR after antiviral therapy and remained HCV-RNA negative both in serum and liver tissue. The presence of HCV-RNA associated with persistence of B-cells bearing chromosomal translocation t(14;18), which is frequently found in B-cell non-Hodgkin’s lymphomas, and mixed cryoglobulinemia syndrome [74]. HCV infection is known to associate with abnormal proliferation of lymphocytes, and residual HCV-RNA was found in 12 out of 32 (37.5%) seronegative patients with malignant lymphoproliferative disorders, who were HCV-RNA negative using standard tests in serum [75]. A similar prevalence of seronegative OCI (27 OCI out of 77–35%) in a group of patients with lymphoproliferative diseases was found by Kisiel et al. [76] Interestingly the majority of HCV-positive patients had HCV-RNA in their bone marrow and in nine individuals, viral persistence was confined only to this compartment, with no signs of infection in PBMC or serum [76].

So far significant associations of OCI with clinical effect were shown in analyses with small groups of patients, and further large-scale studies are needed to confirm these data. To reliably distinguish patients with and without residual HCV-RNA in numerous samples, a reliable method of OCI detection is of primary importance.

## 6. Epidemiological Significance of OCI

Residual HCV-RNA has been detected in populations free of markers of ongoing HCV infection and previous exposure to the virus. Persisting HCV was found in high-risk groups such as injection drug users—9.6–18.2% [77,78], patients with beta-thalassemia major—3.3–6.3% [38,79], hemodialysis patients—4.8–45% [80,81], or infectious liver disease-free subjects undergoing phlebotomy—1.27% [82]. A recent meta-analysis of studies on populations from the Middle East and Eastern Mediterranean countries estimated the pooled rate of OCI in patients diagnosed with cryptogenic liver disease to be 20.8% [83]. OCI was also shown to co-exist with HIV or HBV infections. These viruses share common transmission routes with HCV and co-infections with evident markers of ongoing infection in serum are prevalent in high-risk populations. OCI is frequent in HIV-infected individuals with the prevalence ranging from 9.2% to 11.4%, and reaching 31% in HIV/HBV co-infected patients reported in one study on patients from Georgia [84,85,86]. In addition, simultaneous occult HBV infection and OCI were identified in 1.1% of Iranian HIV-infected individuals [86]. Although in HCV/HBV coinfected liver cells HCV inhibits HBV replication, seronegative OCI was also found in active HBV carriers [82].

Unexpectedly OCI was also identified in an apparently healthy group from the general population—3.3% [87], and random blood donors from Spain, Mexico, and China—0.1–3.4% [88,89,90]. In Egypt, which has one of the highest HCV infection rates, the pooled OCI prevalence estimated by meta-analysis of studies on healthy populations reached 4.79% [83]. In fact, it is possible that OCI can be prevalent in other groups where the presence of HCV-specific T-cell responses without detectable viremia and seroconversion are frequently found, such as healthcare workers, prisoners, household contacts of CHC patients, and their spouses [91]. It was shown that such responses can be hallmarks of not only past exposure and resolution of HCV infection but can also indicate ongoing OCI [45,92].

So far, the potential epidemiological significance of these infections remains to be determined, as there is no data unequivocally confirming transmission of HCV infection from persons with OCI. It is known, however, that residual HCV persisting in patients’ PBMCs is infectious in vivo [93], and in a chimpanzee model [94]. In fact, only 20 copies of the virus were sufficient to transmit HCV infection in chimpanzees [95]. Additionally, the transmission of low levels of HCV can be masked by an over 6-month long eclipse phase where HCV-RNA remains undetectable even with the highly sensitive RT-PCR assay, before establishing a high-level viremia [96]. Other reports show that OCI could be responsible for the observed cases of late relapses of HCV infection in patients who reached SVR after therapy [96,97,98], as well as of donor liver reinfection in successfully treated CHC patients after transplantation [34,99]. Furthermore, it was found that the prevalence of occult infection among family members of patients with OCI is similar to that of relatives of CHC patients [100].

Thus, the risk of HCV transmission from subjects with OCI exists and detection of residual HCV-RNA in PBMC samples of blood donors raises important questions about the safety of blood testing. As the standard screening of blood donations for anti-HCV antibodies, HCV-RNA, and elevated liver enzymes are not sufficient to exclude OCI, it was suggested to include other tests such as detection of anti-HCV core antibody [101,102,103]. Some authors underline the necessity of screening serial samples as HCV-RNA levels seem to fluctuate over time [6,104].

## 7. Inconsistencies in Approach to Detect Occult Infection

The existing studies differ significantly in reporting the prevalence of occult infection. Some authors detect no or very rare cases of OCI, concluding that the clinical significance of this infection is negligible, and questioning the necessity to test for the presence of residual HCV-RNA [105,106,107,108]. Others find a relatively high percentage of OCI [5,53,103]. These discrepancies stem from various methodologies applied to detect viral RNA. Table 1 summarizes some of the recent reports on OCI prevalence in order to underline technical differences in OCI detection strategies and also in the reporting of the key parameters in the HCV-RNA detection procedure (Table 1). The definition of OCI includes no clear-cut boundaries either for the amount of clinical sample for RNA isolation, sensitivity, and strand specificity of assays used for HCV-RNA detection. Without introducing a more unified approach, it is difficult to draw a firm conclusion on the true prevalence of OCI and its real clinical impact.

Below we discuss several points in HCV-RNA detection methodologies that can be a source of bias and that should be considered in future studies and when interpreting the results of OCI detection. Some of those important technical issues have been previously raised by other authors [6,109].

### 7.1. Patients Selection

Differences in criteria of initial patient selection can greatly impede the comparison of OCI prevalence reported by different authors. In some studies, only those patients who have persistently elevated liver enzymes are selected for analysis among the initially enrolled individuals [34,108]. However, as this parameter cannot be regarded as a reliable marker for occult infection [5], the true OCI prevalence in the initial, unselected group of patients, can be underestimated. Others combine seropositive and seronegative individuals for analysis [76,107] while these patients can present two distinct types of OCI with different immunological backgrounds, and various OCI percentages. Viral genotype can be another factor worth considering during the analysis of patients with therapeutically-induced SVR. It was shown that HCV load in PBMC is significantly greater for genotype 1 infected patients than for genotypes 3 and 4 [41]. On the other hand, OCI is more frequent in patients with genotype 3 HCV than in those with genotype 1 [55].

#### Immunosuppression in OCI and CHC

Another important variable in the selection of patients is the presence of immunosuppressive therapy. Some authors argue that if patients had OCI before transplantation or oncological treatment, immunosuppression applied afterward would lead to a robust HCV replication [108,110,111]. Indeed, enhanced HCV replication was observed in CHC patients treated with systemic chemotherapy or immunosuppressive therapy [112,113,114,115,116]. Immunosuppression after liver transplantation seems to stimulate HCV compartmentalization and the emergence of leukotropic variants in chronically infected patients [113]. However, the real impact of immunosuppression and chemotherapy on viral replication during OCI is currently unknown. It is possible that this effect can be quite opposite to what is seen in CHC patients. Such different responses were reported when PBMCs from CHC and OCI patients were ex vivo stimulated with mitogens [10]. Viral eradication was observed in the case of CHC patients while stimulation of HCV replication was evident in PBMCs from individuals with OCI. This phenomenon was found to be strictly dependent on the basal HCV-RNA load in the lymphoid cell compartment [10]. Thus, it cannot be excluded that the levels of residual HCV-RNA in PBMCs of immunosuppressed individuals remain even below detection thresholds of ultrasensitive methods, while withdrawal of treatment could lead to viral replication and a detectable HCV-RNA.

Additionally, various immunosuppressive regimes exhibit a different effect on HCV replication during CHC, including inhibition of viral replication by rapamycin or cyclosporine A [117,118] and stimulation of HCV viral entry to target cells, and thus facilitating the spread of the infection as seen for glucocorticosteroids [119]. Moreover, rituximab-based chemotherapy was found to stimulate viral replication in HCV-infected individuals [115]. The impact of these drugs on HCV biology during OCI remains unknown, but it should be considered as an important variable, and the immunosuppressive regime should be reported in studies on OCI.

**Table 1:** Summary of the methodology of selected reports on the prevalence of occult HCV infection.

Ref.	Study Group	*n*	Patient Material	RNA Isolation Method	HCV-RNA Detection Method	Method LoD	Equivalent of RNA Amount per Reaction	OCI Prevalence
[37]	Seronegative hemodialysis patients	515	PBMC	AccuPrep viral RNA extraction kit	nested RT-PCR, total HCV-RNA, and HCV-RNA (−) strand	ns	ns	95 (18.4%), [58 (11.3%) with HCV-RNA (−)]
[81]	Seronegative hemodialysis patients	62	PBMC,	Automated extraction; QIAamp Viral RNA Mini kit (Qiagen)	qRT-PCR probe-based Artus1HCV-RG RT-PCR Kit (Qiagen)	ns	ns	3 (4.8%)
[120]	Seronegative predialysis patients CrCl < 60 mL/min/1.73 m^2^	91	PBMC, serum	ns	RT-PCR	ns	100ng	15 (16.5%)
[38]	Seronegative patients with beta-thalassemia major	181	PBMC	Proba-NK RNA Extraction Kit (DNA-Technology, Russia)	nested RT-PCR	ns	ns	6 (3.3%)
[79]	Seropositive patients with beta-thalassemia major	48	PBMC, plasma	High Pure Viral Nucleic Acid Kit (Roche Diagnostics)	nested RT-PCR	ns	62.5 ng	3 (6.3%)
[39]	Seronegative hemophilia patients with normal LE	450	PBMC	AccuPrep Viral RNA Extraction Kit (Bioneer Corp., South Korea)	nested RT-PCR	ns	ns	46 (10.2%)
[108]	Liver and/or kidney transplant recipients with SVR to DAAs and elevated LE, immunosuppressants- ns	7	PBMC (*n*=7); liver biopsy (*n*=4)	RNeasy mini kit (Qiagen)	nested RT-PCR (Superscript III one-step PCR)	2 IU/mL (5 copies/mL) for RT-PCR	RNA from 5 × 10^6^ PBMC, or 5–30 mg liver tissue	No OCI
[53]	Patients with SVR to DAAs	42	mitogen-stimulated PBMC	TriReagent (Ambion, USA)	real time RT-PCR, total HCV-RNA and HCV-RNA (−) strand	≤1.5 IU (≤5 vge/µg RNA for RT-PCR	500 ng	31 (74%), [29 (69%) with HCV-RNA (−)]
[54]	Patients with SVR to DAAs	1280	PBMC	Automated extraction (QIAamp1 RNA kits, Qiagen)	qRT-PCR probe-based Artus1HCV-RG RT-PCR Kit (Qiagen)	0.19 IU/μL (0.23 vge/mL) for qRT-PCR	ns	50 (3.9%)
[121]	Patients with SVR to IFN-based therapy	52	whole blood	PAXgene Blood RNA Kit (Qiagen)	nested RT-PCR	0.74 IU/mL of blood	ns	2 (3.8%)
[55]	Immunocompetent patients with SVR (various treatment regimens)	60 DAA 50 IFN 30 SR	paired liver biopsies (*n*=110); PBMC (*n*=89)	RNeasy plus mini kit (Qiagen)	Liver biopsies: RNAscope in situ hybridization; PBMC: qRT-PCR (PCR-fluorescence probing, KHB, China)	ns	ns	9 (15%) DAA 5 (10%) IFN+R 2 (6.7%) SR
[122]	HIV co-infected patients after SVR to anti-HCV therapy	123	serum, PBMC	Automated extraction; PBMC: RNeasy plus universal kit, serum: QIAmp miniElute virus spin kit (Qiagen)	nested RT-digital droplet PCR	ns	ns	1 (0.8%)
[77]	IDUs (seropositive and seronegative) HCV-RNA negative in serum	126	PBMC, plasma	High Pure Viral Nucleic Acid Kit (Roche Diagnostics)	nested RT-PCR	50 IU/mL plasma	125 ng	11 (8.7%)
[89]	Healthy blood donors	1037	plasma, PBMC	QIAamp Viral RNA Mini kit-plasma, RNAeasy kit-PBMC (Qiagen)	nested RT-PCR	ns	ns	35 (3.4%)

CrCl, creatinine clearance; DAA, direct-acting antivirals, IDUs, injection drug users; vge, viral genomic equivalents; IFN, interferon with ribavirin; LE, liver enzymes; LoD, lower limit of detection; ns, not specified; PBMC, peripheral blood mononuclear cells; SR, spontaneous resolution; SVR, sustained virological response.

### 7.2. Type of Sample

The type of patient sample analyzed significantly impacts OCI detection rates. Liver biopsies are considered a gold standard for the diagnosis of OCI [102]. However, due to the low availability of this type of material OCI is usually detected in plasma, serum, and/or PBMCs. Some studies suggest that HCV-RNA may occur in PBMC but not in corresponding plasma or liver tissue samples [5]. It was reported that a combination of HCV-RNA detection in plasma and PBMC with testing for the presence of anti-HCV core antibodies enables reliable diagnosis of OCI without the need for a liver biopsy [102]. Testing for HCV-RNA in PBMC has another advantage over analysis in liver tissue and plasma. It was shown that ex vivo stimulation of PBMC with mitogens significantly augments expression of both HCV-RNA (up to 100-fold) and viral NS3 protein (5-fold). This increase is attributed to stimulation of viral replication rather than enhanced cellular proliferation [123]. Indeed, it was shown that ex vivo mitogen treatment of PBMCs improves detection of residual HCV-RNA in patients with secondary OCI from 30% in unstimulated cells, to 75% in mitogen-treated PBMCs [10]. Thus, testing untreated cells may severely underestimate OCI occurrence in this cellular compartment.

The amount of starting material is crucial for reliable detection of HCV-RNA due to the generally small number of viral particles found in OCI (below 100–200 viral genomic equivalents, vge/mL serum, and up to 100 vge/μg RNA in PBMC or hepatocytes). It was reported that the use of up to 4 mL of ultracentrifuged plasma in comparison to standard isolation from 250 μL of plasma significantly improves HCV-RNA detection [6]. Additionally, plasma was found superior to serum when used for HCV-RNA detection [124]. In the case of PBMCs only around 3% of these cells are positive for HCV-RNA in OCI patients [125]. Moreover, it was shown that HCV-RNA can preferentially reside inside certain immune cell subsets in different patients, and the analysis of total PBMC for HCV-RNA, instead of a particular cellular subpopulation can be not sensitive enough to detect viral persistence [5,10]. Although detecting HCV-RNA in a selected purified subset of lymphoid cells is hardly manageable in the screening of large groups of patients, this fact argues for the maximization of the number of analyzed PBMC for ultrasensitive detection of HCV-RNA.

### 7.3. Testing Serial Samples

Longitudinal studies of patients with OCI showed that levels of HCV-RNA in PBMC and plasma [5], as well as HCV-specific cellular responses [126], can fluctuate in time, becoming undetectable for some period, and again reappearing years after HCV therapy. For this reason, it was suggested that a reliable OCI detection should be based on serial sample testing collected at ca. monthly intervals, and preferably both from plasma and PBMC samples obtained at the same time point [6,104].

### 7.4. Processing Patients Material and RNA Extraction Technique

Given the low amounts of viral genomes present in OCI, variation in handling of patients’ samples, including processing time and storage conditions, can severely impact the amount and quality of isolated RNA that is essential for a reliable HCV-RNA detection. Additionally, RNA isolation methods vary greatly in their efficiency. For a reliable determination of OCI, it is also important to rule out contamination of extracted RNA with DNA as sequences homologous to the 5′-noncoding regions of HCV were identified in DNA isolated from human PBMCs [127].

Genome-length viral dsRNA reservoirs present in hepatocytes of chronically infected patients are difficult to detect with standard reverse-transcription protocols due to their great thermal stability. Klepper et al. found that detection of this dsRNA pool requires heating RNA samples to 106 °C before RT-PCR [33]. On the other hand, Elmasry et al. found OCI with similar amounts of (+) and (−) strands without the application of special protocols for strand separation [34]. These data suggest either different stability of HCV-dsRNA in hepatocytes in CHC and OCI or the existence of other factors impacting strand separation and detection (such as tissue storage or RNA isolation protocol). Stimulation of PBMCs with mitogens resulting in enhanced viral replication could improve the accessibility of HCV dsRNA to detection with standard protocols.

### 7.5. Sensitive Detection Method

The current definition of SVR and HCV-RNA negativity in serum is based on standard clinical tests with LoD of HCV-RNA of ≤0.15 IU/mL. First reports on OCI employed ultrasensitive detection of HCV-RNA with RT-PCR followed by nucleic acid hybridization to recombinant HCV DNA probe [3,5]. The LoD of these detection methods reached ≤3 IU/mL (≤10 vge/mL) in serum, and ≤1.5 IU/μg RNA (≤5 vge/μg RNA) from cells. Currently most widely used detection strategies include nested RT-PCR and real-time RT-PCR. Some authors used isothermal Transcription-Mediated Amplification (TMA) assay combined with an HCV-specific RNA capture method [105,107]. Although this method has LoD of HCV-RNA between 5.3 and 14.4 IU/mL in serum, depending on viral genotype, the question is if the assay is equally applicable to detection of HCV-dsRNA, given the temperature of the TMA reaction is low (40–55 °C) [128]. Unfortunately, the majority of studies on OCI prevalence include no information on the sensitivity of the applied detection method or on how the LoD was determined (Table 1). Moreover, the amount of RNA or its equivalent added to detection reaction is rarely reported and the lack of control of RNA concentration and quality undermines the validity of even the most sensitive HCV-RNA detection method. Insufficient information on detection methodology significantly hampers the interpretation of the results and data comparison between studies.

In summary, we recommend the following key points to be considered in studies on OCI: (i) careful patients’ selection, (ii) maximization of amount material for RNA isolation, (iii) control of RNA amount and quality, and (iv) establishing sensitivity of the HCV-RNA detection method. Additionally, if the specific settings of the study enable, we suggest detection of residual HCV-RNA in at least two compartments, determination of OCI in ex vivo mitogen-stimulated PBMC, detection of both total HCV-RNA and HCV-RNA (−) strand, and testing longitudinal samples from the same patient. In addition, an appropriate description of patients and procedures is absolutely essential for informative and reproducible research on OCI.

## 8. Conclusions

The persistence of low levels of HCV-RNA in PBMC and/or liver, accompanied with undetectable viremia in serum can have important consequences for patient’s clinical outcomes. However, our understanding of the molecular mechanism underlying OCI still remains limited. Considering the rapidly growing group of patients with SVR after DAAs it is important to establish the true OCI prevalence and to determine the trajectory of the disease and its clinical impact by long-term surveillance of individuals diagnosed with OCI. These results could help to establish whether wide-scale screening and special treatment strategies for OCI are needed. In the light of various methodological pitfalls in the detection of residual HCV-RNA, obtaining reliable results of OCI detection will require developing more unified approaches. Further understanding of this disease entity could be facilitated by the identification of new biomarkers of occult infection more suitable for reliable screening of larger sample sizes, and useful in the clinical setting.

## Data Availability

Not applicable.
